# Generating FAIR research data in experimental tribology

**DOI:** 10.1038/s41597-022-01429-9

**Published:** 2022-06-16

**Authors:** Nikolay T. Garabedian, Paul J. Schreiber, Nico Brandt, Philipp Zschumme, Ines L. Blatter, Antje Dollmann, Christian Haug, Daniel Kümmel, Yulong Li, Franziska Meyer, Carina E. Morstein, Julia S. Rau, Manfred Weber, Johannes Schneider, Peter Gumbsch, Michael Selzer, Christian Greiner

**Affiliations:** 1 Institute for Applied Materials (IAM), Karlsruhe Institute of Technology (KIT), Kaiserstrasse 12, 76131 Karlsruhe, Germany; 2 KIT IAM-ZM MicroTribology Center (µTC), Strasse am Forum 5, 76131 Karlsruhe, Germany; 3 Fraunhofer Institute for Mechanics of Materials IWM, Woehlerstrasse 11, 79108 Freiburg, Germany

**Keywords:** Materials science, Mechanical engineering

## Abstract

Solutions for the generation of FAIR (Findable, Accessible, Interoperable, and Reusable) data and metadata in experimental tribology are currently lacking. Nonetheless, FAIR data production is a promising path for implementing scalable data science techniques in tribology, which can lead to a deeper understanding of the phenomena that govern friction and wear. Missing community-wide data standards, and the reliance on custom workflows and equipment are some of the main challenges when it comes to adopting FAIR data practices. This paper, first, outlines a sample framework for scalable generation of FAIR data, and second, delivers a showcase FAIR data package for a pin-on-disk tribological experiment. The resulting curated data, consisting of 2,008 key-value pairs and 1,696 logical axioms, is the result of (1) the close collaboration with developers of a virtual research environment, (2) crowd-sourced controlled vocabulary, (3) ontology building, and (4) numerous – seemingly – small-scale digital tools. Thereby, this paper demonstrates a collection of scalable non-intrusive techniques that extend the life, reliability, and reusability of experimental tribological data beyond typical publication practices.

## Introduction

**Fig. 1:**
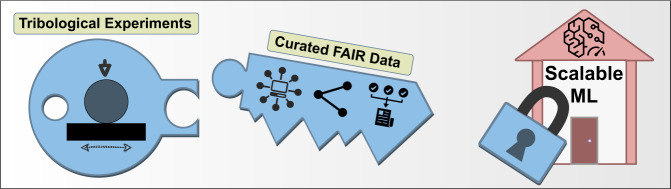
*Visual Abstract*: Unlocking the potential for scalable data science techniques in tribology is only possible through the serial production of FAIR datasets. However, generating truly FAIR data cannot be an afterthought, but rather has to be an integral part and objective of every tribological experiment.

Data are the fundamental asset which attaches value to any scientific investigation. It is not surprising that the expectations of high-quality data, which can travel seamlessly between research groups and infrastructures, are shaping the policies responsible for allocating public funds^1–3^. This drive led to defining the guiding principles that qualify research data as findable, accessible, interoperable, and reusable (FAIR)^4^. Observing these guidelines has since then prompted the creation of detailed metrics^5,6^ that assess whether shared digital objects satisfy these standards and add value for the future users of published data^7,8^. However, the benefits of making data FAIR reach beyond the ease of communication. Increasing data’s trustworthiness^9^ eases the process of transforming data into knowledge^10^ and facilitates its potential utilization by autonomous computer algorithms from the field of machine learning (ML)^11^, as shown conceptually in the visual abstract in Fig. 1.

Generating FAIR research data in tribology is particularly challenging because of the exceptional interdisciplinarity of the field: many seemingly trivial tribological problems require a deep, but still holistic, understanding of processes and mechanisms that act between, at, and underneath contacting surfaces^12^. A tribological response is often regarded as the response of the entire tribological system signifying the importance of all aspects of the actual tribological situation. This complicates the creation of discipline-specific data infrastructures and the standards for experimental procedure and result documentation are still missing^13^. The lack of standards can be partially attributed to the characteristic that tribologists usually interpret research results through the prism of their own scientific backgrounds, which can span a wide variety of physical science and engineering fields^14,15^.

In tribology, the precise sequence of events, and seemingly insignificant external influences, can have a profound effect on the outcomes of any given experiment. Because of that, data provenance is paramount for the generation of knowledge. This extends what “FAIR data” means for tribological experiments: besides the data and metadata generated during the tribological experiment itself, tribologically-FAIR data requires a fully machine-actionable information set of all involved processes and equipment that preceded the tribological test. Previous studies focused on repeatability and reproducibility^16^, including multilaboratory round robins^17–19^, have identified that nominally coordinated tests find good agreement only “*in tribological terms*”^17^ – a standard deviation of 14% qualifies as “*surprisingly low*”^19^. It was shown that, after fundamentally matching the involved specimens^18^, the factors which explain the poor reproducibility include inconsistent tribometry setups^17,19,20^, specimen preparation procedures^16,17,20^, operating conditions^18^, operator’s experience^16^, as well as assumed model^19,21^ and analysis procedures^16,17,19^. One of the paths for using non-aligned results in the quest for demystifying friction and wear’s underlying mechanisms goes through sharing all collected data^22^. A noteworthy example of data publishing in tribology^23^ provides a glimpse into the level of detail expected by the community; however, the metadata and descriptions for the experiments is mostly given in a narrative form (contrary to being machine-readable), and thus fails to qualify as interoperable.

The quest for FAIR data in tribology boils down to the careful synchronization of the following two necessary efforts: (1) to find a schema of categories which generalizes tribological processes and objects, as agreed upon by a critical number of scientists, and (2) a lab framework and digital infrastructure that offers full flexibility of workflows and encourages the recording of both known and previously unaccounted details of the scientific process; this would all be ideally executed at-source and in directly machine-actionable ways (motivating Fig. 1). From a managerial point of view, it is key that the two efforts grow simultaneously as a combined solution to tribology’s digital transformation.

Coping with the challenge of defining the common terms which describe tribological specimens, equipment and data manipulation, aims to: (1) ease the communication between scientists by providing a standard; (2) enable developers of electronic lab notebooks to design suitable user interfaces; and (3) unite existing knowledge to provide computational algorithms a reasoning foundation. Although central to the digitalization effort, to our knowledge, curated metadata repositories, or strategies for the creation of controlled vocabularies, do not exist within the tribological community. For the related field of materials science an example controlled vocabulary took five years to develop - a high-level thesaurus comprising 500 terms^24^. The generality of these terms, however, does not suffice to represent the intricacies of a typical tribological process. Multilateral agreement^25^ on the semantic definitions that describe tribological experiments emerges as one of the bottlenecks in the FAIR transformation of the field, and part of the reason for that is the lack of suitable tools that provide straightforward collaboration in such efforts.

Ontologies provide a scalable framework for knowledge formalization^26^ where domain experts in tribology can traceably encode their expertise in both human- and machine-operable manners. Put more broadly, ontologies represent domain-specific knowledge by defining classes of things (e.g., a tribometer, a human, etc.), their attributes (e.g., a free-text description of what a tribometer is), individuals (e.g., tribometer with a serial number AB12345), and relations between classes (e.g., human *operates* tribometer). Ontologies representing facts based on the Resource Description Framework (RDF)^27^, and providing multifaceted relations and descriptions of the entities that they contain, can be encoded via the Web Ontology Language (OWL)^28^. The resulting graphs not only provide unambiguous knowledge representation that can potentially be used for ML^29^, but most importantly, satisfy many of the FAIR guiding principles. Among them are (“F”) attaching rich metadata, and assigning globally unique and persistent identifiers (through the use of Internationalized Resource Identifiers (IRIs)), (“A”) providing a standardized communication protocol (through the RDF query language called SPARQL), and (“I”) making data and metadata interoperable via the traceable knowledge representation^30^.

Building an ontology for the field of tribology is a nontrivial task, not only because of the already mentioned broad multidisciplinarity of research aspects, but also due to the uniqueness of most experimental procedures, often specific to each laboratory, and custom-built tribometers. A safe starting point for building an ontology is the use of existing higher-level ontologies, which widen the reasoning capabilities within established schemas shared by other ontologies. So far, tribAIn^31^ appears as the only ontology for tribology. One of the aims of tribAIn is to formalize knowledge across the whole field of tribology usually delivered in publications via natural language. However, as the authors acknowledge, organizing already published knowledge bears a high outlay for manual annotation, and eventually only provides the information included by the authors, which most often does not satisfy the FAIR data requirements. Thus, there are two possible approaches to fully harness some of the benefits of an ontology in practice: first, limit the scope of the ontology to narrow and well-defined boundaries which can be controlled for consistency, and second, remove the human intermediator between experiments and recorded metadata by collecting all relevant information at-source.

The technological backbone of the digitalization of an experimental environment is the software infrastructure which acts as a meeting point between controlled vocabularies (organized in an ontology) and the experimental data. Electronic lab notebooks (ELNs) generally offer an environment where researchers can record their observations digitally. However, the choice of an ELN is far from straightforward when the adherence to the FAIR principles as an end-goal becomes a priority. The role of the ELN then is not only to be a digital replacement for handwritten notes, but also to (1) provide an intuitive interface for guiding researchers to the minimum required metadata and ensure their recording with minimal human error (e.g., as predefined key-value pairs), (2) relate records of data and associated metadata, (3) assign unique identifiers to digital objects, and, (4) provide researchers the option to publish their results in data repositories with minimum extra effort^5^. The Karlsruhe Data Infrastructure for Materials Science (Kadi4Mat)^32^, a virtual research environment which includes an ELN, shows a clear commitment to these principles by offering tribologists two major benefits: direct integration with custom tribometers (for at-source data collection) and export of data and metadata in both machine- and user-readable formats.

To assess the feasibility of producing FAIR data via the integration of a controlled vocabulary, an ontology, and an ELN, this paper demonstrates the implementation of a tribological experiment while accounting for as many details as possible. The intricacies of producing such a dataset, at times seemingly administrative (e.g., specimen naming conventions) are equally as important as the global decisions (e.g., using an ontology), in order to provide a reusable pipeline. With this publication, we provide a possible blueprint for FAIR data publication in experimental tribology, and highlight some of the associated challenges and the potential solutions. If applied at large, they may accelerate the rate of innovation in the field and prevent unnecessary and wasteful repetition of experiments. A sister publication^33^ offers the software developer’s point of view and a detailed description of the programmatic backbone of this project.

## Results

### End-to-end framework for FAIR data production

**Fig. 2:**
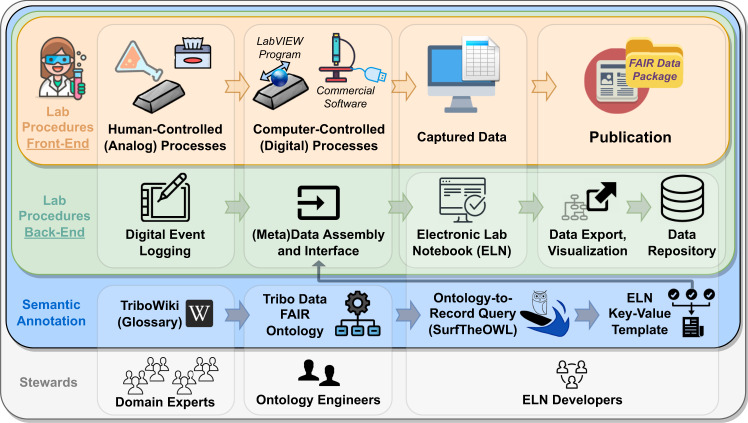
Component diagram of the implemented FAIR data production. The top layer (in orange) contains the front-end, which follows an intuitive digital route to the production of the so-called *FAIR Data Package*. The package’s contents are available on Zenodo^34^ and described in the supplemental video guide (https://youtu.be/xwCpRDnPFvs^35^) while the components of the pipeline are discussed in the Results and Methods sections of the paper.

Producing FAIR data and metadata is not a standalone add-on to the operations of an experimental lab, but rather an integrated collection of scientific, software, and administrative solutions (Fig. 2). Each of the elements in this framework is coordinated with the rest, with the aim of producing a FAIR Data Package^34^. The many groups and sequential routes which Fig. 2 describes stand to show an example of how the different actors (bottom) make their contribution to digitalization (blue and green layer), in order to facilitate the workflow of lab scientists (in orange). The back-end collection of digital tools (in green) can only be effective at communicating with the user if it is provided with the correct knowledge representation (coming from the blue layer).

From a managerial point of view, the ELN (Kadi4Mat^32^) administers the storage of data, the users who interact with it, and the timestamps of its manipulation; in effect this charts a *who-*and-*when* map. At least equally important is the *what*-and-*how* contents of the FAIR Data Package, which originates at the tribological experiment, and is the main focus of this publication. To make the presentation of this multifaceted project most effective, this chapter presents its various components, first describing the FAIR Data Package, which has a clear target composition, and then, the details about its building blocks.

### FAIR data package of a tribological experiment

The driving philosophy of the FAIR Data Package is the availability of detailed descriptions and metadata, in both machine- and human-operable forms. While the standard data structures based on formal notations are aimed to be used by automated computer algorithms, the visually-appealing human-friendly outputs aim to engage the human perceptions and natural intelligence.

**Fig. 3:**
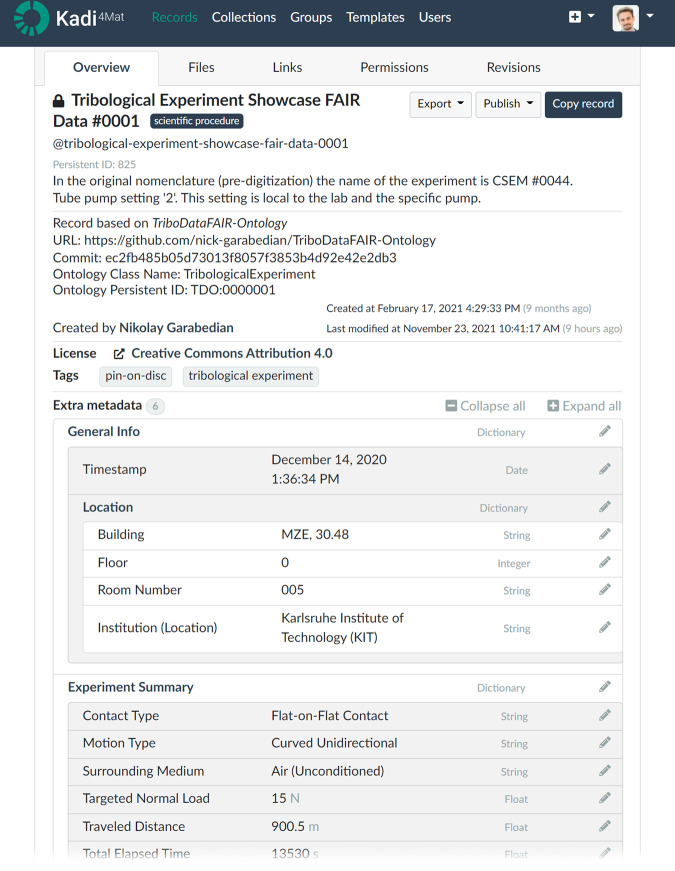
An example of a Kadi4Mat *Record* which includes the *Record*’s own metadata, the entity’s metadata (“Extra metadata”), uploaded *Files*, *Links* to other *Records*, and the access and version controls. This screenshot is only a sample representation, while the published records do not include personal information, such as the creator’s name, which is executed programmatically when exporting the *Collection* of *Records*.

The basic fundamentally distinct information object in the FAIR Data Package is the *Record* (Fig. 3). Each *Record* is stored in the ELN and contains its own metadata (author, last revision/creation time, persistent ID, license, tags, corresponding ontology class – to name a few) and the details of the entity it represents. A *Record* can contain various externally generated data, such as tables, text, images, and videos, but also the *Links* to other *Records* (e.g., a tribometer *Record* is related to a tribological experiment *Record*), or hierarchies of *Records* (e.g., a *Record* is part of a *Collection* which unites all participating entities in a project).

When exported for sharing, a *Record* has two forms: a human-readable PDF and a key-value structured JSON file; in case there were files uploaded, such as raw measured or processed data, a zipped file archive is added. For the showcase experiment performed for this publication, all associated *Records* were grouped in a Kadi4Mat *Collection* and then uploaded to Zenodo^34^ where they were automatically given a digital object identifier (DOI). A noteworthy feature of this export is the ability to anonymize the *Records* before exporting them, so that researchers’ privacy is preserved (more information listed in Table 1). A detailed video guide to the FAIR Data Package is available in video format at https://youtu.be/xwCpRDnPFvs^35^.

**Table 1:** Summary of proposed naming conventions used in the FAIR Data Package. This establishes a lab-wide system for recording information, which speeds up the communication between researchers and the ELN. This table is provided as a set of suggested solutions for other laboratories going through a digital transformation.

Topic	Solution	Reasoning
User Tokens	Uniquely assigned and randomly generated. These four-character tokens are kept in a registry that is administered internally for the lab.	Using this approach, the researchers’ names cannot be uniquely associated with a specific time and place (lab), but individual-specific trends can still be traced.
Specimen Name	Freely chosen by the responsible researchers and kept in a registry, which does not allow repeats.	In this way individual researchers can decide what the most pertinent information to be encoded in the specimens’ name is. As such, specimen names will follow different systems, in order to serve the primary user of the samples best. Of course, this is only in addition to the unique persistent identifiers for each specimen within the ELN.
Record Type within ELN	- lab equipment	These types are for ease of navigation within the ELN. They are sourced from the respective superclass in the ontology for each record, and displayed via SurfTheOWL.
	- industrial procedure	
	- scientific procedure	
	- data processing	
	- experimental object	
Record Name within ELN	Class Friendly Name + Free Name of Choice + Optional Counter	- The *Class Friendly Name* is listed in the relevant class in the ontology.
		- *Free Name of Choice* is only for the users’ convenience, so it could be any one that doesn’t result in repeated record titles.
		- The *Optional Counter* starts with a number sign (#) and is followed by 4-digit sequential number with leading zeros, if there are more than one of the same processes or objects. For example: “Interfacial Medium Shell V 1404 #0001”.

**Fig. 4:**
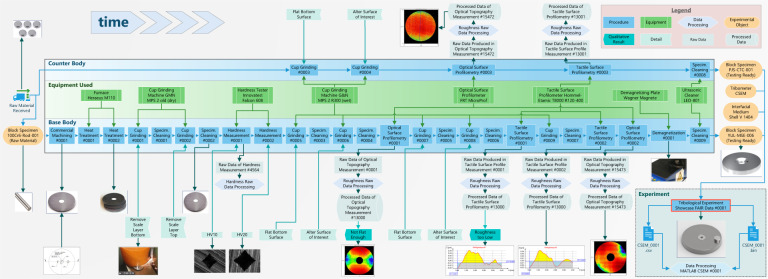
Timeline of the processes and objects comprising the showcase FAIR tribological experiment. Note that the *Experiment* itself occupies only the space in the bottom right corner, and sits at the end of the workflow. However, the experimental workflow preceding the tribological test has to be considered as an essential part of the tribological experiment if FAIR data principles are to be satisfied. Selected visuals from the FAIR Data Package are also included. To distinguish the types of workflow elements they were colored and shaped as: procedures (dark blue rectangle), experiments (green rectangle), data processing (light blue hexagon), experimental objects (orange ellipse), qualitative results (teal pentagon), process detail (light turquoise pentagon), raw data (light turquoise rectangle), processed data (light turquoise cylinder).

**Fig. 5:**
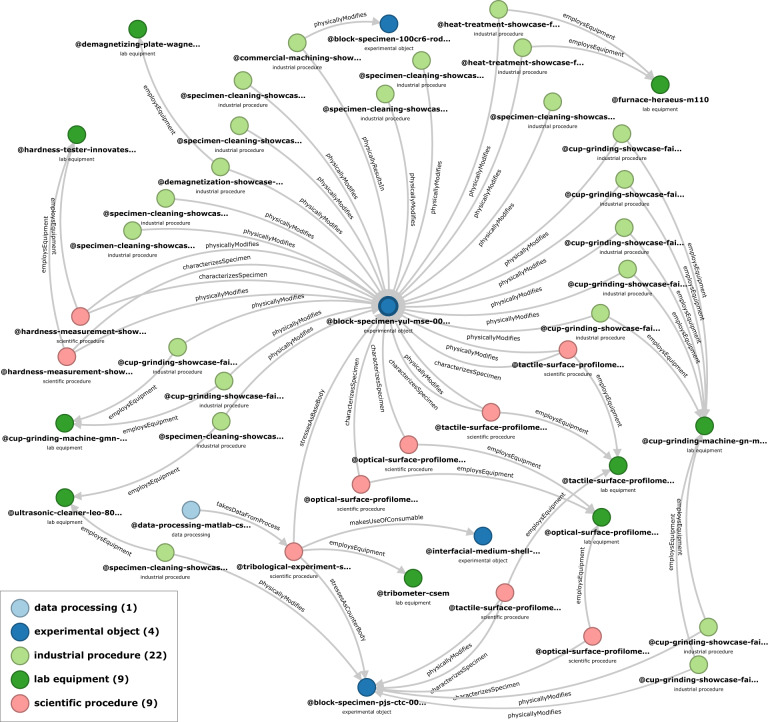
Knowledge graph visualization generated automatically by Kadi4Mat, based on the *Records* which it contains and the *Links* derived from the TriboDataFAIR Ontology^37^. When viewed online, inside Kadi4Mat’s interface, the items in the graph contain the hyperlinks to specific *Records*. The record type (as listed in Table 1) are colored as: data processing (light blue), experimental object (dark blue), industrial procedure (light green), lab equipment (dark green), scientific procedure (red).

The Zenodo repository^34^ also includes two visual summaries which represent two distinct viewpoints: a time-based workflow (Fig. 4) and a links-based ontology-derived graph (Fig. 5). When a workflow is considered, the tribological specimens (base and counter bodies) take a central importance as the carriers of information across the experiments. On the other hand, when the logical links are considered, the tribological experiment is the center of semantic connections. For this publication, the logical links visualization was generated automatically, while the workflow was manually composed as its automatic counterpart is in beta testing. Lastly, the ontology which guides the contents and *Links* of all *Records* is referenced with the URL where it can be downloaded from, with its relevant GitHub “commit hash”.

### Using an ELN in tribology

Kadi4Mat and its ELN was selected because it has as a main objective the production of FAIR data coming from a diverse portfolio of data-producing sources. With the virtual research environment, which automatically records and backs up all necessary data and metadata, tribologists can focus on the procedural details of experiments and pay more attention to previously overlooked characteristics of their workflow.

### At-source production of FAIR metadata

The success of deploying a new system for data and metadata collection within the established workflows of tribological laboratories is at best challenging. Therefore, it is paramount that a FAIR data framework induces minimum disruption to the current research practices (front-end view in Fig. 2). However, implementing a new lab-wide system is also an opportunity to raise the overall efficiency of the lab’s operations. With this in mind, the following two example solutions were developed for two representative processes, as an attempt to bridge the hands-on experimental activities with Kadi4Mat.

**Fig. 6:**
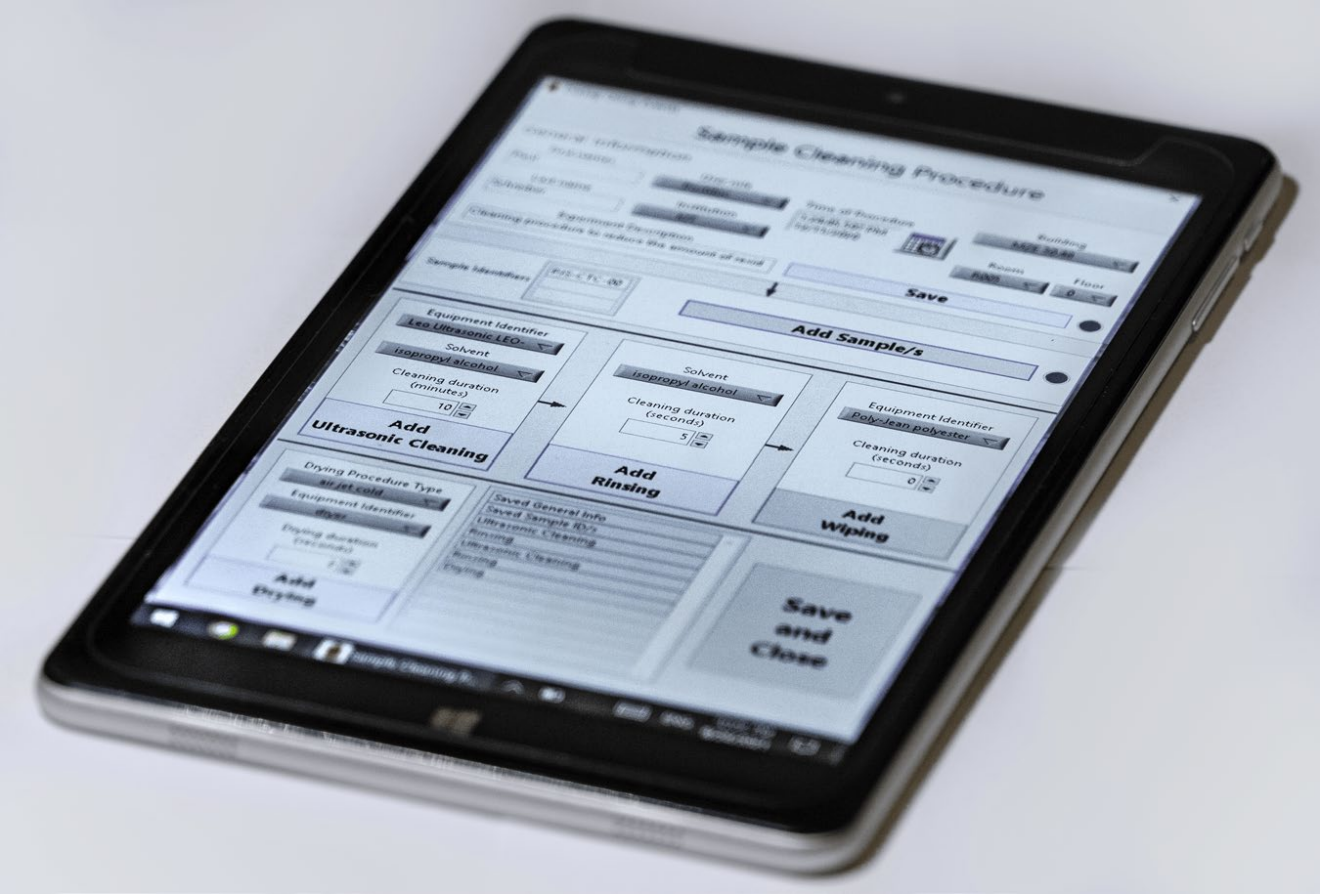
A GUI for collecting predefined key-value descriptions of the event sequence comprising a typical specimen cleaning procedure – a digital interface for an inherently analog process. This digital event logging (also referenced in Fig. 2) can be included in the same information pipeline as computer-controlled processes.

Most of our tribometers are currently “in-house” developments, which test a narrow range of research questions. As such, these tribometers are usually controlled by LabVIEW. Conveniently, this offers access to all data and metadata while they are being collected. To package and upload this information to Kadi4Mat for one of these tribometers, a straightforward code which establishes a connection with the server hosting the ELN was added at the end of the already existing LabVIEW code; the technical details of this procedure are outlined in the sister publication^33^. Such machine-operated processes are in contrast to the “analog” processes in experimental tribology like specimen milling, polishing, cleaning, and storage. These processes do not have files as an output and their details have hitherto only been recorded in paper lab notebooks, without any formalized vocabulary or a system. Thus, the showcase solution that was developed for collecting analog information for specimen cleaning consists of a guided user interface (GUI), which was programmed in LabVIEW and runs as a standalone executable on a tablet computer (Fig. 6). The GUI offers an intuitive way of ensuring that all requisite details are collected in a formalized manner, while in the backend, it allows upload of the assembled *Record* to Kadi4Mat. Critically, the existence of controlled vocabularies will enable the creation of more such GUIs with similar interfaces which will in turn streamline the onboarding of new researchers into the lab.

Removing the intermediary (i.e., the human operator) from the process of metadata collection (wherever possible) has the added benefit of ensuring that the recorded descriptions always comply with the community-agreed standards. In order to bridge the ontology of standards to Kadi4Mat, another module, called SurfTheOWL^36^, was programmed, which assembles the tree of required metadata for each *Record*. SurfTheOWL’s JSON output supplies a machine-operable template for this tree, which can be integrated with Kadi4Mat, while, its counterpart, SurfTheOWL’s Web output composes a human-readable equivalent, which can be used for either validating the ontology’s structure or, in exceptional cases, a backup method of manual creation of ELN *Records* (also mentioned in Fig. 2).

### An ontology of FAIR tribological experiments (TriboDataFAIR Ontology)

The main motivation behind building an ontology for the scope of this showcase experiment was threefold: first, to make the collected data interoperable; second, to provide a scalable environment for metadata manipulation and expansion; third, to support the construction of a knowledge graph based on the collected data^37^. However, before an ontology could be composed, a controlled vocabulary database had to be amassed. As outlined in the methods section, the group of domain experts collaboratively built such a controlled vocabulary, which contained some basic semantics, and ensured to unambiguously describe all entities that reflect typical tribological processes and objects. However, this MediaWiki-based database is nonideal when it comes to scalability and interoperability – two areas that ontologies excel in. The process of transforming the controlled vocabulary into an ontology is nontrivial as it requires extensive linguistic curation: it is essential that the correct terms are used to achieve the best balance between generality and specificity. Furthermore, extensive domain knowledge was needed to build the class hierarchy in an extensible manner. Strategically, before the ontology was initiated, a representative showcase experiment was chosen, which limited the scope of needed terms and provided a clear envelope for the extent of details needed, by asking the question: *Can one redo the same experiment based exclusively on the information in the ontology?*

**Fig. 7:**
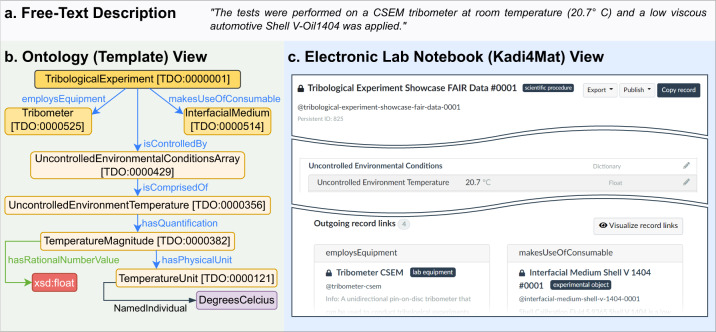
A comparison between different ways of reporting experimental details. (**a**) A typical free-text sentence taken from the Methods section of this paper. (**b**) The formalized ontological representation of the semantics in the sentence. The object properties (in blue and green) direct the way SurfTheOWL assembles the fields that are to be filled out in an ELN. (**c**) The description of the showcase experiment formalized within Kadi4Mat and based on the connections in the TriboDataFAIR Ontology.

The general philosophy of the TriboDataFAIR Ontology^37^ is that procedures utilize, alter, and/or generate objects. For example, a tactile surface profilometry procedure simultaneously characterizes, but also physically modifies specimens. Such interactions between physical objects are subclasses of the object property *involves*. For simplicity and ease of understanding, the ontology models roles as object properties, rather than separate classes, e.g.: counter and base bodies are modeled as object properties. As a result, the ontology does not contain a class “Sample” (a case-specific role of an object), but rather “BlockSpecimen” (the object irrespective of its use). Figure 7 exemplifies how the ontology takes the semantic description of an event and in turn provides a template for its documentation in the ELN. Further, the version control of the ontology is ensured through the use of a GitHub repository (https://github.com/nick-garabedian/TriboDataFAIR-Ontology) and shared persistent identifiers for the classes that are used in the ELN: TriboDataFAIR Ontology with an acronym “TDO”. The TriboDataFAIR Ontology, also listed on FAIRsharing.org (https://fairsharing.org/3597), can easily be expanded to include more complex description logic, but was decided to keep its structure as general as possible, as long as it satisfies its expected competency. The direct use case of the ontology, which also serves as its competency test, is the inclusion of the *Kadi4MatRecord* class; tracing the object properties and subclasses that originate at this class supplies the template (via SurfTheOWL^36^) for the creation of metadata *Records*. The competency question thus becomes: *What keys need to be provided to an ELN, so that after associating each of them with a value, the currently showcased tribological processes and events will be described FAIR’ly?*

### Showcase pin-on-disk experiment

**Fig. 8:**
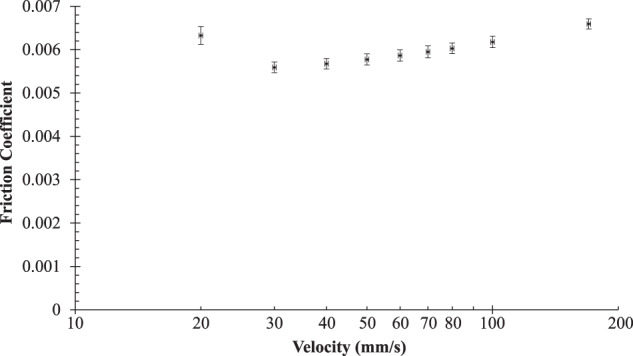
Friction coefficient measurement results of the showcase lubricated pin-on-disk experiment. The range of velocities for this test represent the early stages of hydrodynamic lubrication. The friction coefficient data points and error bars represent the mean and standard deviation, respectively, of the last three out of five consecutive velocity ramps, while the velocity error bars represent the uncertainty of the electrical motor.

For this publication, a showcase pin-on-disk experiment was conducted, while recording all FAIR data and metadata details; hydrodynamic friction results are shown in Fig. 8. Using the infrastructure developed for this project, documenting thorough descriptions required significantly less time and effort than for other procedures in the lab; furthermore, sharing them publicly took only a few short steps, and facilitated their potential exchange and integration into larger future investigations.

The scope and level of detail that descriptions, data, and metadata have to encompass in order to qualify as tribologically FAIR are ultimately up to the discretion of the domain experts composing the FAIR Data Package. Before community standards are firmly established, experienced tribologists have to, at the very least, select the features that make their experiments repeatable and reproducible; in our case three such features are local and global surface topography, magnetization, and hardness – each thoroughly described in their own ELN records.

## Discussion

The FAIR Data Package provided together with this publication was assembled with the aim to observe the FAIR data metrics^5^ as closely as possible; all descriptions and metadata related to all procedures and objects that are involved in implementing the showcase experiment are provided. However, there is still information that was impossible to retrieve, because external suppliers of materials and equipment often do not report details that at first might not seem important; however, as a highly interdisciplinary field, tribology will necessitate these features in the future. The same applies to commercial research software, as already recognized by other groups^38^. Existing datasets from prior publications can also be included in the scheme through text mining techniques based on the ontology, especially as it grows. New but incomplete datasets can also be accommodated by the framework as ontologies offer multiple grades of generality. However, incomplete datasets have lower reasoning weight and, as such, it is ultimately up to the dataset producer to pick the number of details to include, and thus determine the data’s value.

The information that is accessible to tribologists, on the other hand, is often not documented with the necessary depth, although it could be key for future investigations. However, developing the appropriate digital infrastructure (e.g., through an ELN) is not a separate standalone process or a fix-all solution. Rather, it must advance in parallel with the development of controlled vocabularies, ontologies, and most importantly, it has to be in close contact with practicing researchers who can field test them. The exchange of information between the various teams in the digitalization process is easiest to achieve through human-friendly outputs at every step of the way.

Finally, finding a common unified standard that serves the needs of all experimental tribologists in the world seems utopic. However, what can be done is to at least adopt a common framework for metadata creation, which guarantees the interoperability of individual developments, especially through the use of ontologies. Unfortunately, this will involve cross-disciplinary knowledge in the fields of tribology (with its subfields), ontology development, machine learning (for putting the ontology structure in the correct light), and computer science (for developing the data infrastructure); a list of types of expertise which are not readily available in tribology labs. Additionally, the specialized tools needed to accelerate labs’ digital transformation are still under development^39^. We hope that this publication and the showcased solutions will contribute some basic pieces to such a development, will spark the dialogue for this process, and will encourage more participation.

## Methods

The sections in this chapter present the chronological order of execution, in order to provide clarity to the motivations behind the choice of the various tools utilized in this project.

### Conceptualization

The project began by collecting a set of easy-to-identify, well-constrained lab objects, procedures, and datasets. Arguably the most effective way for compiling such а list is through visuals; we used the open-source software Cytoscape^40^ which is a platform originally built for visualizing complex networks and performing network analysis, e.g. on genome interactions based on big biological datasets. Although any other graph-building platform could have been used instead, Cytoscape features an intuitive user interface which let us create the mind-maps needed for the next steps in the project.

Interestingly, even at this early stage, the tribological specimen emerged as the most-important carrier of information in an experiment, as it was also identified by other groups^31^. However, describing the sample itself hardly provides enough information to resolve why a particular tribological phenomenon occurs. In fact, it is the details of the processes and objects that support the preparation of a specimen that can let us explore what the relevant factors are in reproducing a particular set of tribological results – a well-known challenge as described in the introduction.

### Controlled vocabulary

After the standalone entities (objects, processes, and data) were identified in the initial Cytoscape chart, their semantics had to be drafted and agreed upon by the group of domain experts (the ten participants in this part of the project are identified under “Author contributions”). The platform for such an effort had to offer simultaneous editing by multiple users, hierarchical and non-hierarchical structuring of elements, version control, and an intuitive user interface. As a result, a local instance of the MediaWiki^41^ software was installed at the institute and named TriboWiki; in the well-known environment it was relatively easy to manage the collaborative progress through the extensive use of subpages and links. An additional benefit to this solution was the availability of extension modules, as well as, an integrated native API (application programmable interface) which enabled external archiving and manipulation of the contents, for example, for automatic progress visualization in MATLAB. The end result at this stage of the project, after approximately 4 months and 21 group discussion meetings, were the entity descriptions which reflected the synchronized views of all researchers, and a preliminary structure in their relations.

### Showcase experiment

The decision to implement a relatively standard experiment was vital for the success of this project because it prescribed a clear focus and made it more tangible, while helping it stand out from other digitalization efforts. The showcase experiment had a lubricated pin-on-disk arrangement, ran at 15 N normal load and a velocity range of 20 to 170 mm/s. The tests were performed on a CSEM tribometer at room temperature (20.7 °C) and a low viscous automotive Shell V-Oil1404 was applied. The fully detailed technical parameters can be found in the FAIR data package of this paper^34^. While the experimental steps were performed, the contents of the internal TriboWiki were field tested for completeness and appropriateness. When technological solutions were needed to fulfill the FAIR data guidelines and the objectives of this project, the experimental pipeline was paused until suitable solutions, like the ones described in the following paragraphs, were built.

### Ontology development

An Ontology of FAIR Tribological Experiments (called TriboDataFAIR Ontology) was developed to provide both a scalable medium for the showcase-experiment-relevant semantics in the TriboWiki, but also to make the collected descriptions and metadata interoperable. The software called Protégé^42^ was used for the development of the ontology, while SUMO^43^ and EXPO^44^ were used as foundational upper ontologies, and tribAIn^31^ was used to a limited extent when possible. Assembling the contents of the TriboDataFAIR Ontology while conducting the showcase experiment had a two-fold effect: on the one hand, it uncovered the missing gaps in the logical structure of the connections in the TriboWiki, while filtering out any repetitive and ambiguous definitions; on the other hand, the execution of the experiments guaranteed the competency of the ontology in accurately representing the needed objects, processes, and data.

### Electronic lab notebook

Kadi4Mat^32^ was used to capture information at-source and store all collected data and metadata according to the standards established up to this point in the project. The application and evolution of Kadi4Mat for the purposes of collecting FAIR tribological data, are presented in a sister publication^33^. LabVIEW was used to enable at-source generation of descriptions, data, and metadata for a showcase computer-controlled process (*Tribological Experiment*), coupled with an automatic upload to Kadi4Mat, and for the documentation of a sample analog procedure (*Specimen Cleaning*). A Python code using Django^45^ and owlready2^46^ was composed to automatically pull all experiment-necessary information contained in the TriboDataFAIR Ontology and restructure it from a class hierarchy into an intuitive “description/metadata hierarchy” (Fig. 7). Importantly, this latter automated approach serves as a competency test for the ontology and verifies its consistency, as it provides a differently organized view of the contained entities than the inherent class structure, which is easy to verify by a human operator before a process is conducted, and in turn makes the collected metadata interoperable.

## Data Availability

The FAIR Data Package of tribological data can be found on Zenodo^34^, where it is versioned in case of updates. Additionally, the TriboDataFAIR Ontology can be found at https://github.com/nick-garabedian/TriboDataFAIR-Ontology with its most up-to-date changes, while main updates are listed as versions in Zenodo^37^.

## Code availability

The virtual research environment Kadi4Mat, its documentation and source code, can be found at https://kadi.iam-cms.kit.edu/.

The SurfTheOWL application (both source code and standalone executable) for deriving key-value pairs from the TriboDataFAIR Ontology is available at https://github.com/nick-garabedian/SurfTheOWL with its most up-to-date changes, while main updates are listed as versions in Zenodo^36^.
